# Anti-Inflammatory Prostanoids: Focus on the Interactions between Electrophile Signaling and Resolution of Inflammation

**DOI:** 10.1100/tsw.2010.69

**Published:** 2010-04-13

**Authors:** Beatriz Díez-Dacal, Dolores Pérez-Sala

**Affiliations:** Department of Chemical and Physical Biology, Centro de Investigaciones Biológicas, Consejo Superior de Investigaciones Científicas, Madrid, Spain

**Keywords:** cyclopentenone prostaglandins, 15d-PGJ2, PPAR, proteomic studies, cysteine modification, electrophilic lipids, redox regulation

## Abstract

Prostanoids are products of cyclooxygenase biosynthetic pathways and constitute a family of lipidic mediators of widely diverse structures and biological actions. Besides their known proinflammatory role, numerous works have revealed the anti-inflammatory effects of various prostanoids and established their role in the resolution of inflammation. Among these, prostaglandins with cyclopentenone structure (cyPG) are electrophilic lipids that may act through various mechanisms, including the activation of nuclear and membrane receptors and, importantly, direct addition to protein cysteine residues and modification of protein function. Due to their ability to influence cysteine modification–mediated signaling, cyPG may play a critical role in the interplay between redox and inflammatory signaling pathways. Moreover, cellular redox status modulates cyPG addition to proteins; thus, a reciprocal regulation exists between these two factors. After initial controversy, it is becoming clear that endogenous cyPG are generated at concentrations sufficient to promote inflammatory resolution. As for other prostanoids, cyPG effects are highly dependent on context factors and they may exert pro- or anti-inflammatory actions in a cell type–dependent manner, or even biphasic or dual actions in a given cell type or tissue. In light of the growing number of cyPG protein targets identified, cyPG resemble other pleiotropic mediators acting through protein modification. However, their complex structure results in an inter- and intramolecular selectivity of the residues being modified, thus opening the way for structure-activity and drug discovery studies. Detailed characterization of cyPG interactions with cellular proteins will help us to understand their mechanism of action fully and establish their therapeutic potential in inflammation.

## GENERAL ASPECTS OF ANTI-INFLAMMATORY PROSTANOIDS

The term prostanoids refers to lipids derived from 20 carbon fatty acids by the action of the enzymes called cyclooxygenases (COX), namely prostaglandins (PG) and thromboxane (TX). Prostanoids are involved in many pathophysiological processes, including regulation of vascular tone, fertility, platelet function, tumor biology, and inflammation. Fig. 1 presents a schematic summary of the formation of various prostanoids and related lipids. The substrate fatty acids for the action of COX are released from membrane phospholipids by phospholipases. COX are bifunctional enzymes that sequentially generate $PGG_2$ and $PGH_2$ through a two-step reaction involving the cyclooxygenation and peroxidation of the fatty acid substrate, which is mainly arachidonic acid. $PGH_2$ is, in turn, transformed by various synthases into the PG and TX. The specific distribution of the various synthases in precise cell types gives PG synthesis a cell type–dependent character. Thus, whereas $TXA_2$ is the main product of the COX pathway in platelets, $PGE_2$ is synthesized in many different cell types. $PGI_2$, also called prostacyclin, is mainly synthesized in endothelial cells and $PGD_2$ is a major COX-pathway product in various types of cells, including macrophages, dendritic cells, and activated mast cells[1,2]. Two types of PGD synthase exist, known as lipocalin and hematopoietic-PGD synthase, mainly expressed in the central nervous system and immune and inflammatory cells, respectively[3]. In addition, it should be taken into account that COX-2 may act not only on free fatty acids, but also on esters or amides, such as 2-arachidonoylglycerol or anandamide. 2-Arachidonoylglycerol is converted into $PGH_2$ glycerol ester, which can be subsequently transformed by PG synthases to yield $PGE_2$-, $PGF_2$-, or $PGD_2$-glycerol[4], whereas anandamide is transformed into prostanoylethanolamides, such as $PGE_2$ ethanolamide[5]. It is interesting to note that, in some cases, products of aspirin-acetylated COX-2, like 15*R*-HETE, originated in one cell, can be metabolized in a different cell, in a process known as transcellular metabolism, giving rise to 15-epi-lipoxins, which play an important role as proresolution mediators[2,6].

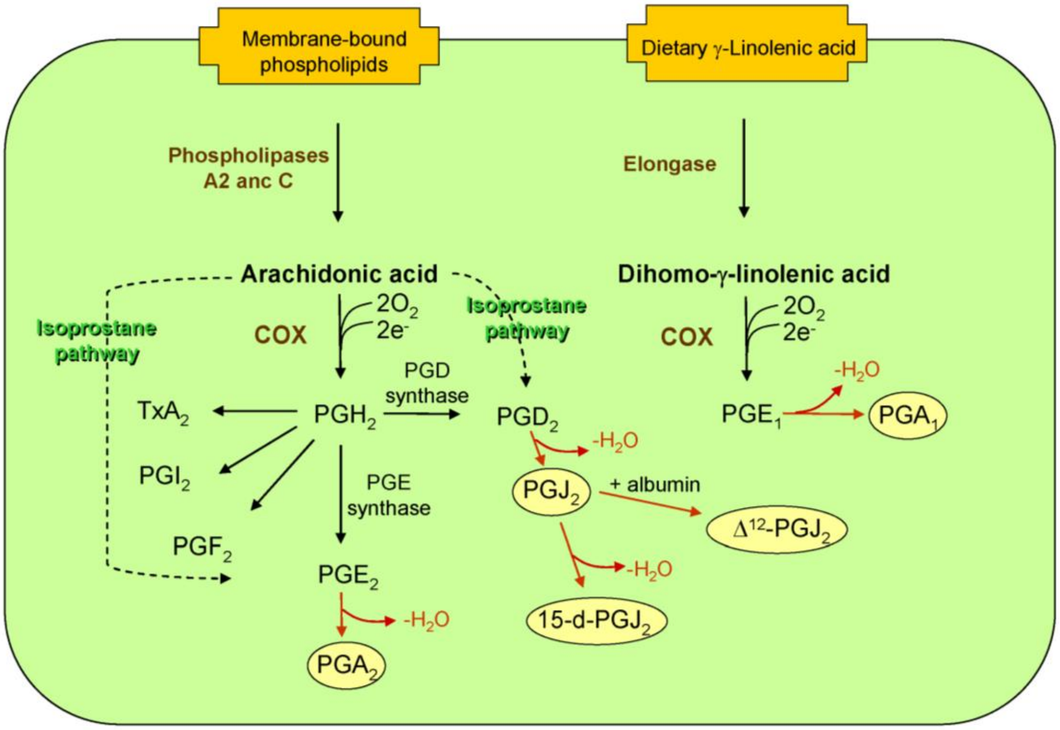

**FIGURE 1.** Pathways for the synthesis of prostanoids. COX enzymes acting on polyunsaturated fatty acids generate PG. The PG common precursor $PGH_2$ generated from arachidonic acid can subsequently be transformed by synthases into the various PG and $TXA_2$. It should be taken into account that nonenzymatic transformation of polyunsaturated fatty acids can also give rise to PG through the isoprostane pathway. In addition, nonenzymatic dehydration of some PG leads to the formation of reactive cyPG (in yellow ellipses).

It is important to note that in addition to enzymatic transformations, PG can be further transformed through nonenzymatic reactions, originating a wide variety of structurally and biologically diverse prostanoids. For instance, levuglandins, which are highly reactive lipids able to induce protein modification and aggregation, can be formed from $PGH_2$ by nonenzymatic rearrangement[7]. Nonenzymatic dehydration of PG can also occur, leading to the formation of PG with cyclopentenone structure (cyPG), electrophilic lipids with potent anti-inflammatory actions[8,9,10]. Finally, although the term prostanoid is used to refer to COX-derived eicosanoids, it should be noted that a substantial amount of $PGD_2$ and $PGE_2$ can be formed nonenzymatically by direct oxidation of arachidonic acid through the isoprostane pathway[11]. This pathway also gives rise to the isoprostanes, isomers of the PG that may display related biological actions[12].

Prostanoids play a critical role in inflammation and for many years, COX enzymes and their products have been considered mainly proinflammatory agents. Evidence from the use of anti-inflammatory drugs and from genetically modified animal models deficient in certain PG synthases or PG receptors have evidenced positive roles of prostanoids in the inflammatory response[13]. Thus, $PGE_2$ induces hyperalgesia, fever, and pleural exudation; $PGD_2$ facilitates allergic inflammation; and $PGI_2$ plays a key role in vasodilatation, inhibits platelet aggregation, and may mediate inflammatory swelling, whereas TX is a prothrombotic, platelet-activating prostanoid. Therefore, COX enzymes and PG synthases have been key targets for the action of anti-inflammatory compounds and great efforts have been devoted to the development of inhibitors. Of the two enzymes of this family, COX-2 is considered the form mainly involved in the pathogenesis of inflammation, given the fact that it is not detectable in most tissues, but its expression is greatly induced by proinflammatory stimuli (reviewed in Pérez-Sala and Lamas[14]). This fact, along with the objective to reduce adverse effects of NSAIDs thought to arise from COX-1 inhibition, spurred the search for COX-2–specific inhibitors. Several of the new compounds obtained through intense research, known generically as the “coxibs”, have produced adverse cardiovascular effects, thus revealing more complex roles of COX-2–derived eicosanoids than previously contemplated[15,16]. These adverse effects, which include heart failure and myocardial infarction, appear to be due to the reduction of vascular synthesis of $PGI_2$, which seems to be largely COX-2 dependent in the vascular system, whereas TX synthesis in platelets, dependent on COX-1 activity, is preserved[16]. This imbalance would create a vasoconstrictive, prothrombotic state.

In a work that contributed to changing the view on COX enzymes, Gilroy et al. proposed a role of COX-2 in inflammatory resolution[17]. In this work, a late peak of $PGD_2$ production and its dehydration product, the cyPG 15-deoxy-$\Delta^{12,14}$-$PGJ_2$ (15d-$PGJ_2$), were observed together with an exacerbating effect of NSAIDs at late stages of the inflammatory response. Later, it was reported that mice deficient in hematopoietic $PGD_2$ synthase show an exaggerated inflammatory response that fails to resolve rapidly[10]. In another study, mice lacking COX-1 or COX-2 showed increased susceptibility to intestinal inflammation, more intense in the case of COX-2–deficient animals, thus suggesting a role for COX-2–derived prostanoids in the defense against inflammation (please see Narumiya[13] for review). These and other evidences help to clarify the view that many prostanoids may exert anti-inflammatory actions. The nature of the action of prostanoids is highly dependent on the cell type or the target tissue, due in part to the type of prostanoid receptors or protein targets present. Thus, the same compound may elicit opposite effects in different locations. PGE- and PGD-type PG are clear examples of this and the molecular bases for their divergent effects in different biological contexts have been reviewed recently[13,18]. $PGE_1$ was early described to ameliorate inflammatory arthritis in animal models[19]. In addition, $PGE_2$ has been proposed to regulate the NF-κB pathway negatively[20], and both positive and negative effects of this PG on inducible nitric oxide synthase (iNOS) induction have been described[14]. The cellular context may also influence the nature of the effects encountered. For instance, in some cellular models, $PGE_2$ may elicit different effects in the presence and in the absence of cytokine stimulation (see Scher and Pillinger[20] for review). Differences in the tissue distribution of $PGE_2$ receptor subtypes underlie many of the tissue-specific effects of this PG[1]. $PGE_2$ acting through EP2 and EP4 receptors exerts relaxant effects, whereas acting through EP1 elicits contraction of vascular smooth muscle[21]. However, even when acting through the same receptor subtype, context is also an important determinant for PG action since, for instance, the same receptor subtype, EP4, has been reported to mediate anti-inflammatory actions in intestinal inflammation and proinflammatory effects in arthritis[13].

$PGD_2$, in turn, has been reported to facilitate allergic reactions. However, $PGD_2$ or its derivatives, in particular 15d-$PGJ_2$, have been reported to contribute to the resolution of Th1-driven delayed-type hypersensitivity reactions[22]. Two types of $PGD_2$ receptors exist, DP1 and DP2, which signal through different mediators and may trigger very different cellular responses. Whereas DP2 is involved in leukocyte chemotaxis and allergic inflammation[13], DP1 has been involved both in the development of asthmatic responses and in the negative modulation of inflammation[10].

Regarding cyPG, recent evidence indicates they may also play opposing roles in different settings. Thus, whereas anti-inflammatory effects have been reported in most experimental systems, cyPG may elicit proinflammatory effects in precise situations.

In summary, given their varied effects, a detailed knowledge of the factors that influence PG actions is very important in order to predict potential undesirable effects both of PG and of the pharmacological inhibition of their synthesis. In this line, the development of novel therapeutics based on the endogenous mechanisms of inflammatory resolution and on the identification of novel functions of lipid mediators is a thriving field of research.

## ELECTROPHILIC PROSTANOIDS: THE CYCLOPENTENONE PROSTAGLANDINS

### Formation of cyPG

cyPG are generated by nonenzymatic dehydration of their parent PG. A-series cyPG arise from the dehydration of PGE-type PG, whereas cyPG of the J series are generated by dehydration of $PGD_2$ (Fig. 1). A pathway for the formation of $PGA_1$ in skin has been reported[23]. The pathway for the formation of cyPG of the J series has been elucidated in detail. $PGJ_2$ arises from the spontaneous dehydration of $PGD_2$. In turn, $PGJ_2$ can be further dehydrated to yield $\Delta^{12}$-$PGJ_2$ in an albumin-dependent manner or 15d-$PGJ_2$ in an albumin-independent manner[24]. cyPG were detected in various biological fluids or found to be generated *in vitro* more than 3 decades ago[25,26]. Synthesis of cyPG (reviewed in Noyori and Suzuki[27]) allowed the exploration of their biological effects, which unveiled potent antiviral and antiproliferative actions[28,29]. Structurally, cyPG are characterized by the presence of an $\alpha,\beta$-unsaturated carbonyl group in the cyclopentane ring (cyclopentenone) (Fig. 2). This structure confers a strong electrophilicity to the carbon(s) in the $\beta$-position, which may suffer the nucleophilic attack from nucleophiles, mainly the sulfur atom in thiol groups, giving rise to Michael adducts. Thus, conjugates of cyPG with glutathione (GSH) or with proteins are generated. From the early studies with cyPG, it was realized that the cyclopentenone moiety was a key structure for the activity of these compounds[30].

### Biological Actions

As stated above, the effects of cyPG that were pursued earlier were the **antiproliferative effects**, for which they were envisaged as potential anticancer agents. cyPG were found to increase the life span of tumor-bearing mice and to inhibit the growth of several transformed cell lines[29,31]. The cellular mechanisms involved in the antiproliferative effects of cyPG may be multiple and dependent on the biological system under study. cyPG have been shown to induce apoptosis or cell cycle arrest in association with modulation of cell cycle regulatory proteins, such as cyclins D1 and B1[32,33], cyclin A– and cyclin E–dependent kinases[34], or cyclin-dependent kinase inhibitors[35]. Induction of apoptosis by cyPG or cyPG analogs may also result from mitochondria and/or NADPH oxidase–derived oxidative stress[36]. CyPG have also been shown to modulate prosurvival and proapoptotic factors. Inhibition of the prosurvival factor NF-κB has been proposed to play a role in cyPG-induced apoptosis in several settings[37],

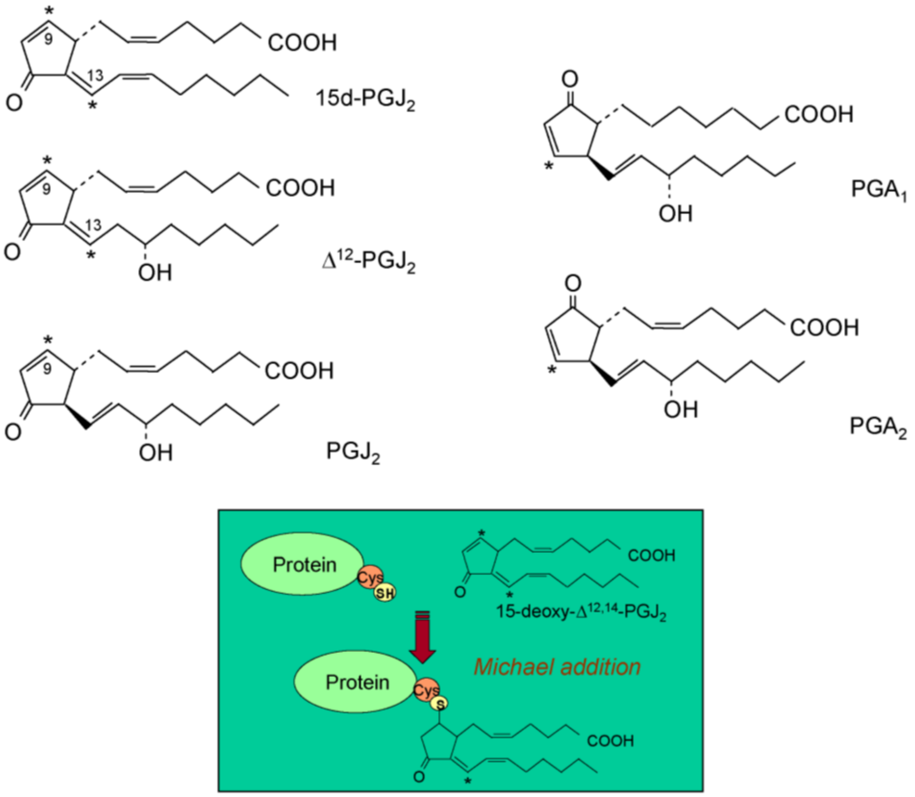

**FIGURE 2**. Structures of several cyPG and scheme of the Michael addition. The structure of several cyPG of the J or the A series is depicted. Note the variability in the configuration and presence of substituents in the lateral chains. Also, the number of electrophilic carbons, marked by asterisks, is different. The lower scheme depicts the adduct formed by nucleophilic attack on the carbon in the β-position of the cyPG.

whereas both positive and negative effects of cyPG on the proapoptotic factor p53 have been reported[38,39,40]. In addition, cytoskeletal disruption[41,42,43], inhibition of protein synthesis[44], or down-regulation of telomerase reverse transcriptase (hTERT) could play an important role in the antiproliferative effects of cyPG[43,45]. Nowadays, the effect of cyPG on cell proliferation has been exhaustively studied in a wide variety of experimental models. Although an antiproliferative effect of cyPG has been described in most settings, it should be noted that cyPG may also induce cell proliferation and/or protection from apoptosis through several mechanisms under various experimental conditions. Protection of keratinocytes from apoptosis induced by the carcinogen DMBA has been proposed to be at the basis of an increase in cell survival after mutagenic injury and, therefore, of a potentiation of tumor progression[46]. Moreover, a protective effect of 15d-$PGJ_2$ on apoptosis induced by $H_2O_2$ has been observed in PC12 cells[47], associated with the induction of the antioxidant response. In hepatic cells, both potentially beneficial and adverse effects of cyPG may take place since 15d-$PGJ_2$ has been found to reduce the fibrogenic response of human hepatoma cells[48], but enhance the toxicity of allyl alcohol and its active metabolite acrolein in isolated hepatocytes[49].

Early studies on the biological activity of cyPG also revealed that they are potent **antiviral** agents. This effect was attributed to their ability to inhibit NF-κB, which is necessary for the replication of several viruses, or to the induction of a heat shock response, which elicits the expression of cytoprotective enzymes[50]. More recently it has been shown that certain cyPG may directly target viral proteins involved in transcription[51]. Thus, the mechanisms involved in the antiviral effects of cyPG may depend on both cellular and viral targets.

The studies on the antiviral effects of cyPG, and in particular of $PGA_1$, led to a wealth of knowledge on the cellular effects of these compounds. Inhibition of NF-κB[52], a transcription factor involved in the induction of numerous proinflammatory genes, was noted more than 10 years ago. Since then, the **anti-inflammatory** actions of cyPG have been explored in cellular and animal models of inflammation. 15d-$PGJ_2$ was shown to inhibit the production of monocyte inflammatory cytokines[53], and to reduce the expression of iNOS in cerebellar granule cells[54] and in glial cells[55]. 15d-$PGJ_2$ also inhibits COX-2 and ICAM-1 induction by cytokines[8], as well as the expression and activity of matrix metalloproteinases (MMPs)[56]. In addition, potentiation of activated macrophage apoptosis by 15d-$PGJ_2$ was proposed to contribute to the resolution of inflammation[57], whereas reduction of endothelial cell-monocyte interaction[58] and decrease in neutrophil migration[59] provided a mechanism for 15d-$PGJ_2$–mediated inhibition of leukocyte recruitment to the lesion site. In animal models, cyPG have been shown to attenuate the development of acute and chronic inflammation[60], to reduce systemic inflammation in polymicrobial sepsis[61], and to reduce edema[62]. 15d-$PGJ_2$ may also reduce other inflammatory symptoms, such as inflammatory hypernociception[63]. Recently, the use of $PGD_2$ synthase knock-out animals indicates that endogenous generation of 15d-$PGJ_2$ plays an important role in limiting the duration of the inflammatory process[10]. Inhibition of proinflammatory transcription factors, such as NF-κB and AP-1, may play a role in the anti-inflammatory actions observed *in vitro* and *in vivo*[64]. Similarly, activation of the antioxidant response and induction of heme oxygenase-1 (HO-1) has been proposed to mediate the anti-inflammatory effects of 15d-$PGJ_2$ in some experimental systems[65]. Indeed, the induction of HO-1 itself has anti-inflammatory consequences, in part through the production of CO and of bilirubin and biliverdin, which have antioxidant properties. In addition, activation of the transcription factor PPARγ underlies some of the *in vivo* reported effects, including attenuation of inflammation in a model of experimental stress-induced inflammation[66]. This is due in part to the ability of PPARγ to interfere with NF-κB and AP-1 function. Therefore, although the mechanisms of action involved may be multiple and are not completely understood, it is clear that pharmacological doses of cyPG attenuate acute and chronic inflammation in several experimental models[60,62,67].

## Mechanisms of Action

From the early studies with cyPG, it was recognized that the cyclopentenone structure and the ability of these compounds to modify thiol groups covalently was important for their actions. Binding of radioactive cyPG to intracellular structures, likely proteins, was evidenced in works by Narumiya and Fukushima exploring the subcellular distribution of cyPG[68]. In 1995, the interest in the biological effects of cyPG was greatly spurred by the finding that 15d-$PGJ_2$ could act as a ligand for the transcription factor PPARγ[69], a member of the nuclear receptor family that plays an important role in the regulation of lipid metabolism and adipocyte differentiation, and that is also emerging as a key regulator of the inflammatory response in some cell types[70]. Although it was not recognized at the beginning, it is now clear that 15d-$PGJ_2$ and other endogenous fatty acid derivatives that act as PPARγ ligands form a covalent adduct with a cysteine residue present in the ligand-binding domain of the factor, cysteine 285, and that this interaction is important to elicit the active conformation of the receptor[71,72].

Shortly after the connection between cyPG and PPAR activation was proposed, many works addressed the potential PPAR-dependent or -independent nature of the effects of 15d-$PGJ_2$. Several approaches have been employed, including comparison of the effects of cyPG with those of other PPAR agonists, such as rosiglitazone or BRL49653, considered highly selective PPARγ agonists, assessment of the ability of PPAR antagonists to block cyPG effects, and comparison of cyPG actions with the effects of other compounds with different ability to modify proteins covalently. Several works can be cited that outline this classical approach[8,73], a schematic view of which is presented in Fig. 3. The involvement of PPARγ in the effects of cyPG and other agonists has also been studied in cells from conditional knock-outs, given the fact that the PPARγ knock-out is lethal (see Barak and Kim[74] for review). Recently,

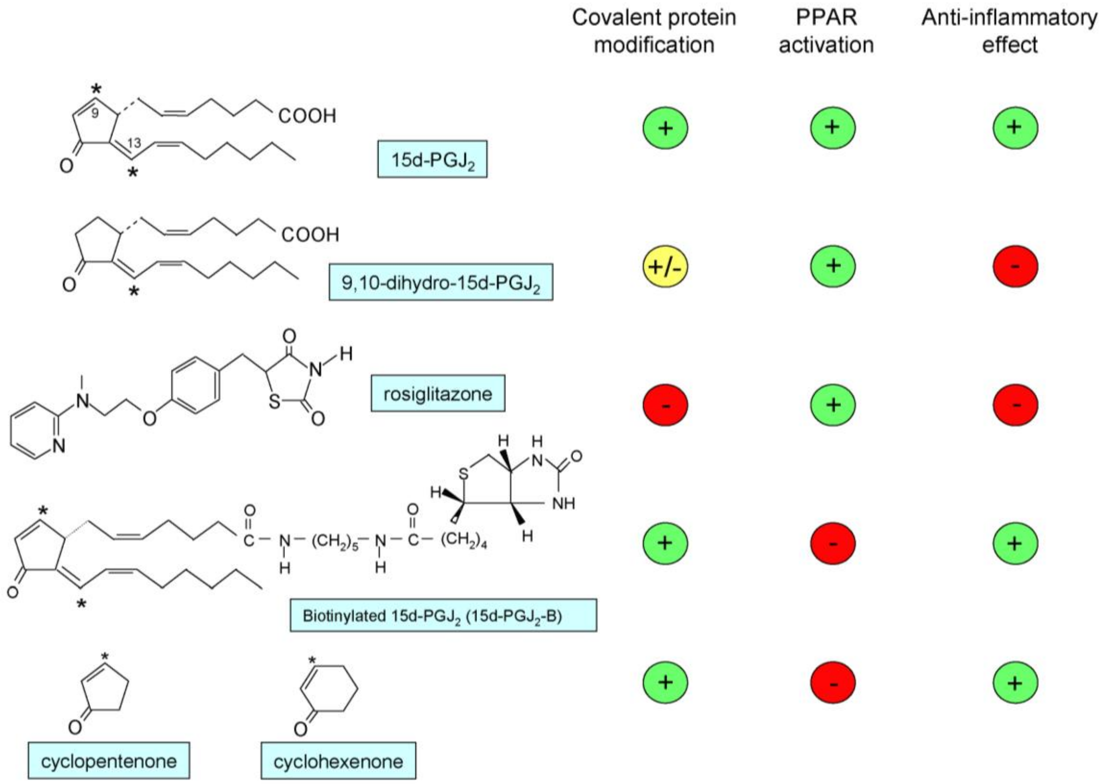

**FIGURE 3.** Contribution of covalent protein modification to the anti-inflammatory effects of cyPG in mesangial cells. A summary of the results obtained in rat mesangial cells through the use of various compounds differing in their ability to activate PPAR and to modify proteins covalently is shown along with their structure. 15d-$PGJ_2$ efficiently activates PPAR, binds covalently to proteins, and elicits anti-inflammatory actions. The analog of 15d-$PGJ_2$, 9,10-dihydro-15d-$PGJ_2$, which lacks the endocyclic double bond, but is a potent PPARγ agonist, shows greatly reduced ability to inhibit the induction of COX-2 or iNOS by cytokines in cellular models. The same applies to rosiglitazone, which is a potent PPARγ agonist, but does not bind covalently to proteins and does not display anti-inflammatory actions in this system. In contrast, simple cyclic compounds with α,β-unsaturated carbonyls, such as cyclopentenone and cyclohexenone, which are inert as PPAR agonists, display anti-inflammatory effects. Biotinylated cyPG bind covalently to proteins and mimic many of the effects of their parent PG in spite of the presence of the bulky biotin moiety, including disruption of the vimentin cytoskeleton, inhibition of iNOS induction by cytokines or induction of permeability transition in mitochondria. Interestingly, biotinylated cyPG appear to be inert as PPAR agonists. Taken together, these observations highlight the importance of covalent protein modification over PPAR activation for the anti-inflammatory effects of cyPG in mesangial cells.

depletion of PPARγ by RNAi techniques has also been used[75]. In these and other works, it has been realized that not only cyPG, but also other PPARγ agonists, may display PPAR-independent effects, which implies that caution should be exercised when interpreting the results of the pharmacological studies in the PPAR field.

cyPG may also interact with membrane receptors. The DP2 receptor is a chemotactic receptor present in various types of leukocytes, which has been involved in allergic inflammation[18]. Several cyPG of the J series bind to this receptor with dissociation constants in the nanomolar range[76]. Interaction of cyPG with DP2 results in calcium mobilization and chemotaxis of leukocytes. There is no evidence for covalent binding of cyPG to DP2. However, cyPG may covalently modify other membrane receptors, such as the ion channel TPRA1, resulting in activation[77].

As research on cyPG progresses, it becomes clear that these prostanoids may covalently bind to multiple cellular targets. Identification of the targets for protein modification by cyPG, some of which appear in Fig. 4, has greatly helped the elucidation of their mechanism of action. The potent anti-inflammatory actions of cyPG rely on the modification of key components of proinflammatory signaling

| Protein | CyPG | Residue modified | Functional consequence | Reference |
|---|---|---|---|---|
| IKKα and β | 15d-$PGJ_2$<br>$PGA_1$ | Cys179 | Inhibition | Rossi et al. 2000<br>Castrillo et al. 2000 |
| NF-κB p65 subunit | 15d-$PGJ_2$ | Cys38 | Inhibition | Straus et al. 2000 |
| NF-κB p50 subunit | 15d-$PGJ_2$ | Cys62 | Inhibition | Cernuda-Morollón et al. 2001 |
| Proteasome | 15d-$PGJ_2$ | Not identified | Inhibition | Shibata et al. 2003 |
| H-Ras | 15d-$PGJ_2$<br>$PGA_1$ | Cys184 or Cys118 | Activation | Oliva et al. 2003<br>Renedo et al., 2007 |
| Thioredoxin | 15d-$PGJ_2$ | Cys35 and Cys69 | Inhibition | Shibata et al. 2003 |
| Thioredoxin reductase | $PGA_1$ | Not identified | Inhibition | Moos et al. 2003 |
| Keap1 | 15d-$PGJ_2$ | Several | Inhibition | Levonen et al. 2004<br>Kobayashi et al. 2009 |
| c-Jun | 15d-$PGJ_2$ | Cys269 | Inhibition | Pérez-Sala et al. 2003 |
| c-Fos | 15d-$PGJ_2$ | Not identified | Inhibition | Pérez-Sala et al. 2003 |
| Actin | 15d-$PGJ_2$ | Cys374 | Structural alteration | Gayarre et al., 2006<br>Aldini et al., 2007 |
| ER-α | 15d-$PGJ_2$ | Cys227 and Cys240 | Inhibition | Kim et al., 2007 |
| Vimentin | 15d-$PGJ_2$<br>$PGA_1$ | Cys328 | Filament collapse | Stamatakis et al., 2006<br>Gharbi et al., 2007 |
| Tubulin | 15d-$PGJ_2$<br>$PGA_1$ | Several | Structural alteration | Stamatakis et al., 2006<br>Gharbi et al., 2007 |
| TRPA 1 | 15d-$PGJ_2$ | Cys421 and Cys621 | Activation | Takahashi et al., 2008 |
| eIF-4A I | 15d-$PGJ_2$<br>$PGA_1$ | Cys264 | Inhibition | Gharbi et al., 2007<br>Aldini et al., 2007<br>Kim et al., 2007 |

**FIGURE 4.** Summary of several protein targets for covalent modification by cyPG. Among the protein targets for covalent modification by cyPG characterized are transcription factors, receptors, and proteins involved in redox control and in protein synthesis and degradation, among others[73,81,141].

pathways. The NF-κB pathway is activated in response to numerous proinflammatory stimuli and mediates the transcriptional induction of genes such as iNOS, COX-2, MMPs, and cytokines. cyPG targets along this pathway include IKKβ, one of the components of the kinase complex that phosphorylates the NF-κB inhibitory subunit IκB, triggering its proteasomal degradation, both the p65 and p50 subunits of NF-κB, and components of the proteasome[78,79,80,81,82]. Thus, cyPG may inhibit IκB phosphorylation and degradation, which results in a blockade of NF-κB nuclear translocation, but they may also directly inhibit NF-κB DNA binding through addition to cysteines present in the DNA binding domain of NF-κB subunits (see Stamatakis and Pérez-Sala[41] for review). cyPG interact at various levels with the signaling pathways controlling AP-1 activation and, in this case, a direct interaction with AP-1 proteins has also been evidenced that results in inhibition of DNA binding[83]. Importantly, research in recent years has clearly established the role of cyPG in the stimulation of the antioxidant response, through the covalent modification of Keap1 and the subsequent activation of

Nrf2[84]. Keap1 is a cysteine-rich protein that serves as a sensor for multiple cysteine-modifying agents, redox, and electrophilic stress. Keap1 acts as an adaptor for a ubiquitin ligase and is responsible for the proteasomal degradation of Nrf2. Modification of Keap1 impairs Nrf2 degradation by an as-yet-not-completely-elucidated mechanism, leading to Nrf2 accumulation and nuclear translocation[85]. This factor stimulates the transcription of genes that possess an antioxidant-response element (ARE) in their promoters, among which are enzymes for GSH synthesis, HO-1, and GST enzymes (reviewed in Nguyen et al.[86]). Evidence gathered from many recent works has led to propose a protective role for Nrf2 in inflammation[87]. Interestingly, a correlation has been found between the potency of several electrophilic compounds of diverse origin as inducers of phase II enzymes and their potency in the suppression of inflammation[88]. Moreover, Nrf2 has been proposed to act as a mediator of the anti-inflammatory effects of 15d-$PGJ_2$[89]. In summary, Nrf2 and its target genes could play an important role in the resolution of inflammation.

In addition to direct modification by cyPG, redox-sensitive cellular proteins and, in particular, transcription factors can be regulated by cyPG through indirect mechanisms. Oxidation-reduction cycles are key for the function of several transcription factors, including NF-κB, AP-1, p53, and Nrf2. Regarding NF-κB, for instance, oxidation events in the cytoplasm may contribute to activation, whereas DNA binding requires the factor to be in a reduced state. The thioredoxin/thioredoxin reductase (Trx/TR) system plays a key role in the reduction of transcription factors. In the case of NF-κB, Trx-1 in the cytosol can interfere with NF-κB activation and nuclear translocation, whereas in the nucleus it enhances NF-κB activity. The regulation of nuclear redox signaling has been reviewed recently in detail[90]. In the nucleus, Trx-1 can reduce transcription factors either directly or indirectly, through the action of a multifunctional protein known as APEX or Ref-1. In addition to their ability to induce oxidative stress in cells through various mechanisms, cyPG may also directly modify and inhibit both Trx[73] and TR[39], thus impairing the capacity of this system to regenerate the reduced forms of transcription factors. It is clear then that in inflammation, a situation where both oxidative stress and generation of cyPG occur, the possibilities for regulation of transcription factor function are multiple. Understanding these complex interactions may open new avenues for therapeutic intervention in the future.

## SELECTIVITY OF PROTEIN MODIFICATION BY cyPG, INTERACTIONS WITH GSH AND INDUCTION OF PROTEIN CROSS-LINKING

cyPG modify multiple cellular proteins. To date, around 100 potential or confirmed targets for cyPG modification have been identified in various studies[43,91,92,93,94,95]. Thus, cyPG are emerging as pleiotropic mediators with multiple effectors and actions. Analogies can be found between cyPG and other mediators able to modify proteins, such as HNE and reactive oxygen or nitrogen species (ROS, RNS). In some cases, the cysteine residues targeted by NO or glutathiolation have also been found to be modified by certain cyPG. This is the case for C62 in p50, C269 in c-Jun, or C118 in H-Ras[96]. Efforts have been devoted to find a consensus sequence for nitrosylation, and recent works outline the importance of a neighboring acid-base motif[97]. In the case of cyPG, it has been proposed that cysteine residues with a low pKa would be preferentially modified, and that an acidic pH in the vicinity of the adduct would contribute to its stability. In addition, steric factors may contribute to defining the binding sites. cyPG are structurally very diverse and they differ in the position of the α,β-unsaturated carbonyl groups, in the structure and orientation of the side chains, and in the presence of one or more electrophilic carbons; thus not all cyPG can accommodate in the same protein-binding sites. Therefore, in the complex scenario of protein modification by cyPG, there is room for selectivity arising from the structural features of both the protein and the cyPG. Indeed, research in recent years has evidenced that protein modification by cyPG does not occur randomly. The pattern of cyPG-modified proteins in a given cell type is clearly different from the pattern of proteins modified by general cysteine reagents, such as biotinylated iodoacetamide, or from that given by total protein staining[8]. Moreover, the proportion in which cellular proteins are modified may be different among cell types. Whereas actin has been shown to be the main target for cyPG addition in SH-SY5Y neuroblastoma cells[43], only a minor proportion of total cellular actin appears to bind cyPG in NIH-3T3 fibroblasts[94]. The wide structural variety of cyPG provides excellent tools to carry out structure-activity studies. In a study with two different biotinylated cyPG, we showed by two-dimensional electrophoresis that the subsets of proteins modified by each biotinylated analog included common, but also distinct, targets[98]. Moreover, cyPG with different structures may target different residues in a given protein. The first examples of this type of selectivity were the H-Ras proteins, in which we showed that 15d-$PGJ_2$ binding required the presence of C184, located in the C-terminal peptide of H-Ras[96], whereas $PGA_1$ bound preferentially to the cysteine located in the GTP-binding site (C118). The existence of three Ras proteins with highly homologous sequences, but diverging at the C-terminal hypervariable domain, allowed us to prove that 15d-$PGJ_2$ bound selectively to H-Ras, which is the only Ras protein with a cysteine residue at position 184, whereas $PGA_1$ could bind to the three Ras proteins since C118 is present in all of them. Ras protein modification correlated with activation of Ras-dependent pathways. Therefore, this study showed that the selectivity of protein modification by cyPG can be both inter- and intramolecular, and result in differential biological consequences[99]. More recently, it has been shown that 15d-$PGJ_2$ and $PGA_2$ target both common and specific cysteine residues of Keap1, although it has been postulated that the common cysteine target (C273) is the sensor for these electrophilic compounds leading to the activation of Nrf2[100].

One important structural feature of cyPG is the presence of one or more electrophilic carbons, resulting from the presence of one or more double bonds conjugated with the carbonyl group (see Fig. 2). cyPG with dienone structure may covalently modify two cysteine residues in the same or in different proteins, leading to intra- or intermolecular cross-linking. Whereas in the first case important conformational changes of the protein can occur, the second possibility may lead to the formation of oligomeric species or to protein aggregates. The first protein for which intermolecular cross-linking by dienone cyPG was observed was the transcription factor c-Jun[83]. More recently, oligomerization and inactivation of citidyltransferase by 15d-$PGJ_2$ has been proposed to result from cysteine cross-bridging[101]. Taken together, these observations indicate that protein modification by cyPG displays structural selectivity, in which both protein and cyPG structural features play an important role. In this setting, it would be important to identify factors that may influence the selectivity of protein modification by cyPG and, consequently, the biological effects of these lipid mediators.

In a physiological context, the presence of GSH can be an important factor modulating the selectivity of protein modification. GSH is the main, low-molecular-weight antioxidant molecule in cells and it can be present at millimolar concentrations. cyPG may form adducts with GSH by enzymatic and nonenzymatic mechanisms, and the kinetics and the stability of adduct formation is different depending on the structure of the cyPG[102]. This, in turn, affects the availability of cyPG for protein modification. This results in protein modification by a single enone such as $PGA_1$ being more dependent on cellular GSH levels than modification by a dienone cyPG such as 15d-$PGJ_2$[98]. Moreover, the modulation of the binding of cyPG brought about by GSH is not uniform for all polypeptides, thus resulting in different patterns of modified proteins. Therefore, GSH levels influence both the extent and the selectivity of protein modification by cyPG.

GST enzymes catalyze the conjugation of GSH with cyPG, the resulting GSH-cyPG conjugates being exported from cells by multidrug-resistance transporters[103,104]. GSH concentrations and/or GST activity may also influence the subcellular distribution of cyPG[27,80]. Thus, in cells with high cytosolic GST activity or GSH content, cyPG has been reported to act preferentially in the nucleus[80]. By using biotinylated analogs of cyPG, we have observed that subcellular distribution of cyPG is indeed cell-type dependent and it correlates with a compartmentalization of the biological effect. In NIH-3T3 fibroblasts, $PGA_1$-B– and 15d-$PGJ_2$-B–modified structures were clearly more abundant in the cytosolic than in the nuclear compartment, as assessed both by fluorescence microscopy and subcellular fractionation[94] (Gayarre et al., unpublished data). In contrast, mesangial cells[8] and RAW264.7 murine macrophages (unpublished observations) showed an even distribution of 15d-$PGJ_2$-B, whereas nuclear accumulation of $PGA_1$-B[94] and 15d-$PGJ_2$-B[105] was observed in HeLa cells. These results correlate with the preferential site of action of cyPG with respect to the inhibition of NF-κB activation, a paradigmatic pathway for cyPG action[64,79,80,81]. As detailed above, cyPG may inhibit NF-κB at several levels, from the cytosolic signaling steps involving IKK-mediated IκB phosphorylation and subsequent proteasome degradation to the direct blocking of the binding of NF-κB subunits to DNA in the nucleus, these events involving the modification of critical cysteine residues in these proteins. Remarkably, in cells showing an even cytosolic/nuclear distribution of biotinylated cyPG targets, such as mesangial cells or macrophages, both cytosolic and nuclear inhibitory events are inhibited by cyPG[64,80]. In contrast, in cells showing preferential nuclear cyPG accumulation, the cytosolic events of NF-κB activation, which lead to IκB degradation and NF-κB nuclear translocation, remain unaffected, whereas DNA binding is clearly inhibited[81]. A nuclear site for cyPG action has also been reported in the case of microglia[55]. Although further work is needed to establish this point unequivocally, these results suggest that modulating cytosolic and/or nuclear GSH content or cyPG detoxifying activity may constitute a strategy to direct the site of cyPG action.

## EFFECTS OF ENDOGENOUS cyPG AND DUAL ACTIONS

Unequivocally establishing the role of cyPG as pathophysiological mediators requires assessing the effects of the endogenously generated cyPG and solving the discrepancies between the concentrations measured in biological systems and those required to elicit biological effects.

It is worth noting that although 15d-$PGJ_2$ has been the first endogenous compound proposed to act as a ligand of PPARγ, the dissociation constants measured in most experimental settings are in the micromolar range. Since in most works, picomolar or nanomolar concentrations of cyPG have been measured, the true pathophysiological role of 15d-$PGJ_2$ as PPAR agonist is under debate. The same applies to many of the anti-inflammatory actions, which have been observed only with micromolar concentrations of these compounds. Assessing the real concentrations of cyPG in biological systems is not an easy task. This is due in part to their high reactivity by which they are rapidly bound to serum or intracellular proteins[106]. Thus, the free concentrations of cyPG measured may be an underestimation of the real levels. One of the first attempts to determine 15d-$PGJ_2$ levels showed increased generation of this cyPG at late stages of the inflammatory process, leading the authors to propose its role in the resolution of inflammation[17]. Recently, another work by this group established this point more clearly by showing increased 15d-$PGJ_2$ levels in inflammatory exudates of wild-type, but not of $PGD_2$ synthase, knock-out animals[10]. Results in this work point to a role of 15d-$PGJ_2$ in modulating the balance of pro- vs. anti-inflammatory cytokines, and regulating leukocyte influx and monocyte-derived macrophage efflux from the inflamed peritoneal cavity to draining lymph nodes leading to resolution.

Importantly, 15d-$PGJ_2$ has been detected in humans. 15d-$PGJ_2$ was immunodetected in the motor neurons of patients with sporadic amyotrophic lateral sclerosis[107]. 15d-$PGJ_2$ has been also detected in joint synovial fluid taken from osteoarthritis or rheumatoid arthritis patients[108], and has been shown to induce chondrocyte apoptosis *in vitro*, for which a pathogenic role of 15d-$PGJ_2$ has been suggested. Conversely, in a study by Blanco et al., plasma levels of 15d-$PGJ_2$, measured by an enzyme immunoassay, were found to be increased in patients with acute stroke with respect to healthy controls[109]. However, in this case, levels of 15d-$PGJ_2$ in the group of patients with atherothrombotic infarcts were found to be associated with good outcome and smaller infarct volume, thus suggesting a neuroprotective effect of 15d-$PGJ_2$ in this condition.

A summary of the concentrations of cyPG detected in various experimental systems or in patients[108,109,110,111,112] is depicted in Fig. 5.

While the precise assessment of endogenous cyPG levels requires further efforts, it is becoming clear that low concentrations of cyPG may also exert important biological effects in experimental settings. Repeated addition of nanomolar concentrations of 15d-$PGJ_2$ result in accumulation of cyPG-protein adducts over time, leading to biological effects equivalent to those obtained by treating cells with a single

| $[15d\text{-}PGJ_2]$ | Sample | Methodology | Reference |
|---|---|---|---|
| 0-300 pg/ml | Synovial fluid from osteoarthritis or rheumatoid arthritis patients | ELISA | Shan et al. 2004 |
| 0.5-5 ng/ml | Cell free peritoneal inflammatory exudates (mice) | LC-MS/MS and EIA | Rajakariar et al. 2007 |
| 800-1000 pg/ml | Cell free inflammatory exudates from carrageenin-induced pleurisy (rats) | EIA | Gilroy et al. 1999 |
| 300 pg/mg | Serum (mice) | ELISA | Vunta et al. 2007 |
| 20 ng/mg | Adipocytes in culture during maturation phase (3T3-L1 cells) | ELISA | Mazid et al. 2006 |
| 60.5 pg/ml | Human plasma from acute ischemic stroke patients | ELISA | Blanco et al. 2005 |
| 1600 pg/mg | Stressed brain cortex (rats) | EIA | García-Bueno et al. 2005 |

**FIGURE 5.** Concentrations of $15d\text{-}PGJ_2$ measured in different experimental systems and in patients.

addition of $15d\text{-}PGJ_2$ at micromolar concentrations[106]. Nanomolar concentrations of $15d\text{-}PGJ_2$ have been reported to increase proliferation of COX-depleted colorectal cancer cells[113]. Moreover, PPAR activation by nanomolar concentrations of $15d\text{-}PGJ_2$ has been reported to inhibit $PGE_2$ production in amnion cells[114] and to enhance B-cell proliferation, differentiation, and antibody production[115]. In addition, nanomolar concentrations of cyPG acting through the DP2 receptor are able to induce eosinophil chemotaxis[116].

As outlined above, although antiproliferative and anti-inflammatory activities of cyPG are most frequently observed, cyPG may induce the opposite effects in different biological systems. Moreover, in several experimental systems, biphasic or dual effects of cyPG have been described that may occur in a concentration- or time-dependent manner. A dual effect of cyPG on cell proliferation has been observed in several cell types[117]. In endothelial cells, biphasic effects on cell viability occur in association with biphasic effects on GSH synthesis[118], with the increase in GSH levels being cytoprotective and GSH depletion resulting in cytotoxicity. Interestingly, other inflammatory mediators acting through alteration of cellular redox status or modification of protein cysteines have been found to exert biphasic or dual effects on various cellular parameters. For instance, NO induces a biphasic regulation of NF-$\kappa$B activity that is associated with a dual regulation of iNOS and COX-2 expression in a time-dependent manner in mesangial cells[119,120]. Interestingly, we have observed that the cyPG $15d\text{-}PGJ_2$ may elicit a dual regulation of iNOS in mesangial cells in a concentration-dependent manner (unpublished observations). Similarly, $PGA_1$ may induce a biphasic regulation of COX-2 induction by IL-1$\beta$ plus TNF-$\alpha$ in the same system[94]. Thus, hypothetically, cyPG could contribute both to the onset and to the resolution of inflammation.

## THERAPEUTIC POTENTIAL AND STRUCTURE-ACTIVITY STUDIES OF cyPG

An ample number of studies have evaluated the effects of cyPG administration in various *in vivo* models of inflammation or tissue damage, and their potential use as therapeutic agents. The antiproliferative potential of cyPG has led to their evaluation as anticancer agents, and some cyPG or cyPG-derived compounds have been the subject of extensive preclinical trials. Several formulations containing $PGA_1$ derivatives have been found to suppress the growth of human ovarian cancer and colon cells inoculated into nude mice[121,122]. Moreover, some cyPG derivatives have been found to inhibit proliferation of cancer cells resistant to other antitumoral agents[121,123]. For these reasons, efforts are devoted to finding cyPG derivatives with increased stability or potency (Fig. 6)[121,123,124,125,126]. Nevertheless, as stated above, assessment of the antitumoral role of cyPG for every particular tumor type is needed since potentiation of tumor formation in response to cyPG treatment has been observed in some experimental systems in association with increased angiogenesis or protection from apoptosis[46].

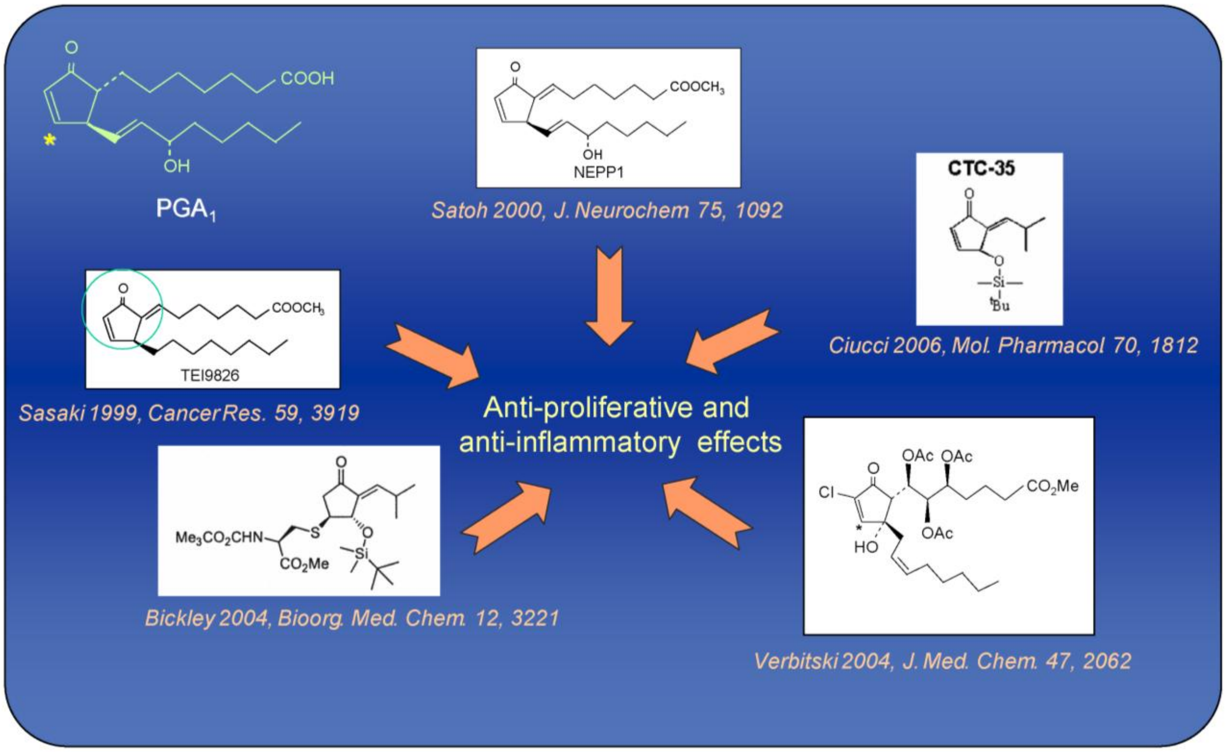

**FIGURE 6.** Several compounds bearing a $PGA_1$-like reactive moiety that have been studied as potential anti-inflammatory or antiproliferative agents.

Numerous studies have addressed the effects of cyPG on tissue injury, and protective effects of these compounds have been evidenced in models of ischemic acute renal failure in rats[127] and ischemia/reperfusion injury of the gut[128], among others, which may be due in part to their direct anti-inflammatory actions or to their ability to activate PPARγ. Beneficial effects of cyPG on the cardiovascular system have been evidenced in models of restenosis after angioplasty[129] and of oxidized, low-density, lipoprotein-induced vascular smooth muscle damage[130]. Moreover, a recent formulation based on the structure of $PGA_2$ has shown promising effects on the symptoms of atherosclerosis in animal models[131].

Although beneficial effects have been observed in most cases, some recent studies call attention to potential adverse effects of cyPG. In the nervous system, in particular, both neuroprotective and neurotoxic actions of cyPG have been reported[132,133]. The identification of cellular targets for cyPG may provide valuable information to understand or even predict potential adverse effects of these compounds. In addition, given the selectivity of protein modification by cyPG, the wide structural variety of cyPG offers the opportunity of performing structure-activity studies and choosing cyPG that modify a particular subset of cellular targets. Molecular modeling or crystallographic studies may help in understanding the molecular basis for this selectivity, and may serve in the design of compounds targeting specific proteins in order to elicit pharmacological or therapeutic actions.

Biotinylated analogs of cyPG have been widely used in the identification of cyPG targets. Our recent studies have shown that biotinylated cyPG mimic many of the effects of cyPG in cellular models, including inhibition of iNOS and COX-2, and induction of HO-1 and Hsp70 expression, but they are unable to elicit PPAR activation *in vitro* or in intact cells[94,134]. Therefore, by adding a bulky moiety to the carboxyl group of cyPG, it may be possible to dissociate some biological actions from the PPAR-agonist activity. This may help to elucidate whether activation of PPAR is an important component of the *in vivo* protective effects of cyPG.

From a more general perspective, the study of protein modification by electrophilic lipids can be important in pharmacology and nutrition. Some active principles present in the diet, such as omega-3 fatty acids and phytoprostanes, are taken up by the organism where they are oxidized, giving rise to electrophilic species (neuroprostanes) with a reactivity similar to that of cyPG[135,136] and may exert anti-inflammatory actions through the covalent modification of proteins involved in proinflammatory signaling, such as IKK[137]. Nowadays there is a growing interest in the study of active principles from natural products. Among them, we could mention various unsaturated fatty acids, polyphenols, such as epillogatecin-galate (present in green tea), curcumin (curry), parthenolide, kamebakaurin, sulforaphane (present in broccoli), isoflavones, such as genistein (soy bean), etc. (reviewed in Surh[138]), for many of which clinical trials have been conducted (please see http://clinicaltrials.gov/ for detailed information). Anti-inflammatory, antioxidant, and antiproliferative or carcinogen-sensitizing effects have been reported for these compounds, many of which possess reactivity towards thiol groups and can form adducts with cysteines in proteins. For many of these compounds, covalent modification of targets along the NF-κB pathway has been reported (see Stamatakis and Pérez-Sala[41] for review). The identification of the protein targets for these compounds could contribute to the understanding of their mechanism of action. From this point of view, the approaches developed in the cyPG field will help in the study of the effects of chemoprotective compounds of natural origin acting through electrophilic mechanisms.

## CONCLUDING REMARKS: CROSS-TALK BETWEEN CELLULAR REDOX STATUS, PROTEIN MODIFICATION BY cyPG, AND INFLAMMATION

As it can be inferred from the works reviewed above, cyPG and other electrophilic lipidic mediators are contributing to the unveiling of the close interactions that exist between redox and inflammatory signaling. Fig. 7 presents a summary of some of these interactions. The modification of cysteine residues plays a critical role in this cross-talk and important efforts are being devoted to the characterization of the so-called “sulfoproteome”, both regarding the nature of the protein targets and the quantitative aspects of cysteine modification. It is clear that cyPG potently alter cellular redox status by multiple mechanisms. cyPG may induce potent oxidative stress by acting on mitochondrial proteins and on superoxide-generating systems[36]. Oxidative stress, in turn, may induce secondary protein modifications, such as thiolation[139]. cyPG can also directly modify and impair proteins involved in the regulation of redox balance and in the regeneration of redox-sensitive function, such as Trx or TR[39,73], thus affecting other redox-responsive proteins indirectly, including transcription factors. Modulation of transcription factors, in turn, may widely influence protein expression patterns. For instance, NF-κB modulation may affect the expression of proteins involved in the generation of RNS, such as iNOS. In addition, cyPG may modulate GSH synthesis through activation of Nrf2-dependent transcription that induces the expression of γ-glutamyl-cysteine ligase, the rate limiting enzyme in this process[140]. However, the complex actions of cyPG on cellular redox status do not constitute a one-way effect. As shown above, the cellular redox status and the availability of antioxidant molecules, mainly GSH, clearly influence protein modification by cyPG through various mechanisms. It is obvious that irreversible oxidation of cysteine residues under conditions of strong oxidative stress would reduce the availability of free thiol groups for potential modification by cyPG addition. Moreover, other reactive species may target the same cysteine residues than these electrophilic lipids through nitrosylation or thiolation. The pathways for cyPG generation, both

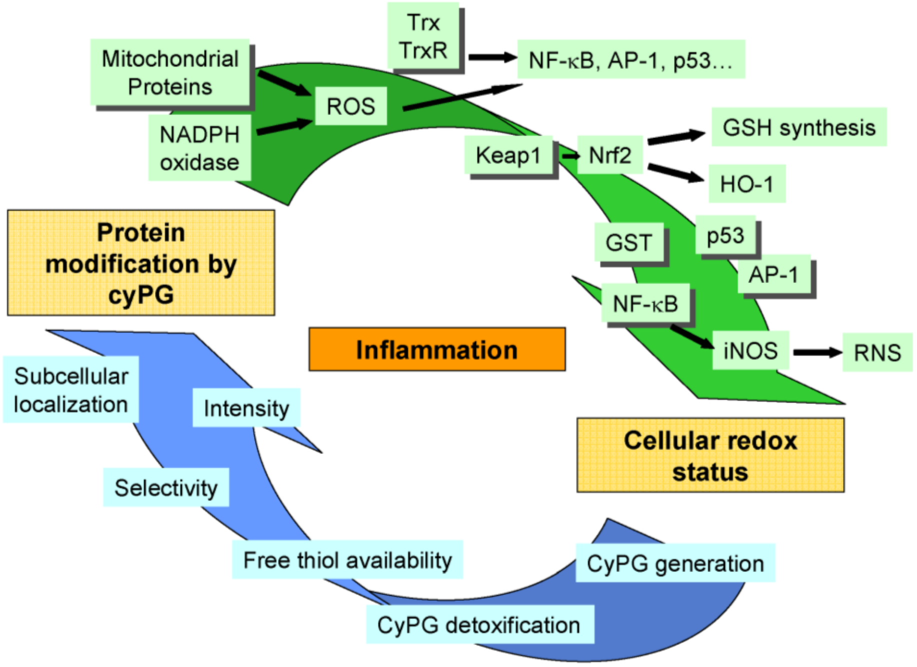

**FIGURE 7.** Reciprocal regulation between cellular redox status and protein modification by cyPG. Proteins shown in shaded boxes represent direct targets for cyPG addition. The function of various cyPG targets, including redox-sensitive transcription factors, such as NF-κB, AP-1, or p53, can be regulated by cyPG through multiple mechanisms (see text).

enzymatic and nonenzymatic, are also subject to regulation depending on the redox status of the cells. COX-2 induction, for instance, is highly dependent on the function of redox-sensitive transcription factors, such as NF-κB or AP-1. GSH content would also influence the cyPG detoxification capacity of the cells, since cyPG-GSH adducts can be exported by multidrug transporters[103]. Importantly, GSH levels influence the intensity and selectivity of protein modification by cyPG[98]. Finally, as outlined above, GSH content may be an important factor in determining the main site of action of cyPG at the subcellular level. Therefore, the effects of cyPG on inflammation and, more precisely, on inflammatory resolution could be finely tuned by the cross-talk with the redox signaling mechanisms.

## ACKNOWLEDGMENTS

We would like to thank Dr. Joan Clarià for editing this review article. This work has been supported by grants from Ministerio de Ciencia e Innovación SAF2006-03489, SAF2009-11642, and RETIC RIRAAF, RD07/0064/0007 from ISCIII.
